# Self-modulation of rectus femoris reflex excitability in humans

**DOI:** 10.1038/s41598-023-34709-4

**Published:** 2023-05-19

**Authors:** Kyoungsoon Kim, Tunc Akbas, Robert Lee, Kathleen Manella, James Sulzer

**Affiliations:** 1 University of Texas at Austin, Austin, TX USA; 2 Intel Corporation, Hillsboro, OR USA; 3 St. David’s Medical Center, Austin, TX USA; 4 Nova Southeastern University, Clearwater, FL USA; 5 MetroHealth Hospital and Case Western Reserve University, Cleveland, OH USA

**Keywords:** Neuroscience, Neurology

## Abstract

Hyperreflexia is common after neurological injury such as stroke, yet clinical interventions have had mixed success. Our previous research has shown that hyperreflexia of the rectus femoris (RF) during pre-swing is closely associated with reduced swing phase knee flexion in those with post-stroke Stiff-Knee gait (SKG). Thus, reduction of RF hyperreflexia may improve walking function in those with post-stroke SKG. A non-pharmacological procedure for reducing hyperreflexia has emerged based on operant conditioning of H-reflex, an electrical analog of the spinal stretch reflex. It is currently unknown whether operant conditioning can be applied to the RF. This feasibility study trained 7 participants (5 neurologically intact, 2 post-stroke) to down-condition the RF H-reflex using visual feedback. We found an overall decrease in average RF H-reflex amplitude among all 7 participants (44% drop, p < 0.001, paired t-test), of which the post-stroke individuals contributed (49% drop). We observed a generalized training effect across quadriceps muscles. Post-stroke individuals exhibited improvements in peak knee-flexion velocity, reflex excitability during walking, and clinical measures of spasticity. These outcomes provide promising initial results that operant RF H-reflex conditioning is feasible, encouraging expansion to post-stroke individuals. This procedure could provide a targeted alternative in spasticity management.

## Introduction

Stiff-Knee gait (SKG) after stroke is a common disability often defined by decreased knee flexion angle during the swing phase of walking^1^. Those with SKG suffer from joint pain, energy inefficiency and increased risk of falls^2^. The causes of post-stroke SKG are unclear, although it has been suggested that quadriceps spasticity contributes to SKG^1^. Our recent work demonstrates this claim, showing an association between hyperreflexia, a component of spasticity, in the rectus femoris (RF) and reduced knee flexion angle in those with post-stroke SKG^3^. Hyperreflexia is defined as overactive or overresponsive reflex activity^4,5^, possibly resulting from disinhibition of brainstem pathways such as the cortico-reticulospinal tract^6–9^ that in turn disinhibit spinal reflex pathways. Thus, a reduction of RF hyperreflexia may help restore healthy gait through restoration of spinal inhibitory pathways in those with post-stroke SKG.

Various pharmacological and surgical interventions have been used to treat quadriceps spasticity. Botulinum neurotoxin (BoNT) injection blocks neurotransmitter release at the neuromuscular junction, resulting in increased range of motion (ROM) in the knee of under 10°^8–11^. BoNT injections alleviate hyperreflexia^9–11^, but also weaken the quadriceps throughout the gait cycle, resulting in limited clinical benefit in those with SKG^9,12^. Surgical interventions such as tendon transfer surgery are also possible, but there is limited evidence for clinical effectiveness^13^. Baclofen, a gamma-Aminobutyric acid (GABA) agonist, is the most common oral treatment for spasticity, however its effectiveness is questionable and results in side effects such as sedation^14,15^. Baclofen is more effective when administered intrathecally in a targeted manner^16,17^, however it may negatively affect compensatory motor systems and requires a surgical implant with regular maintenance. While pharmacological and surgical interventions for hyperreflexia exist, they all have significant drawbacks.

A possible new non-pharmacological, non-surgical procedure could help target hyperreflexia using operant conditioning. Operant conditioning is a type of associative learning process through which the strength of a behavior is modified by reinforcement or punishment^18^. Operant H-reflex conditioning allows the patient to self-modulate their own monosynaptic spinal reflex activity elicited via constant current electrical stimulation of a peripheral nerve (i.e. H-reflex) in a static^19^ or dynamic posture^20^. The typical procedure involves visual feedback of H-reflex amplitude while the participant operantly learns how to control the feedback signal over many training sessions. There is no universally accepted explicit strategy for operant H-reflex conditioning, and it is likely that implicit learning is a major component. Initial work in animal models shows that operant H-reflex conditioning can modulate descending activity of the corticospinal tract and thereby induce spinal cord plasticity that restores altered motor function^21–23^. At present, operant H-reflex conditioning has shown that individuals with spinal cord injury can learn to self-modulate soleus reflex excitability, inducing plasticity at the spinal level^20,24,25^. Further, the ability to operantly condition soleus H-reflex correlates with improvements in walking speed, locomotor symmetry, locomotor EMG activity, and H-reflex modulation during locomotion. For instance, Thompson and colleagues^25^ found that some people with SCI can operantly down-condition the soleus H-reflex to a degree comparable to neurologically intact individuals. Manella et al.^24^ found people with motor-incomplete SCI can modulate reflex excitability through operant conditioning in both directions (up/down) on agonist/antagonist pair (tibialis anterior/soleus). This training resulted in improved walking function on a group level. While operant H-reflex conditioning shows promise for addressing hyperreflexia, it is unclear whether quadriceps H-reflex can be successfully trained, and further, whether those post-stroke can down-condition reflex excitability given potential damage to the corticospinal tract^26^.

In this study, our goal was to examine the feasibility of operant down-conditioning of the RF H-reflex in a pilot cohort including both healthy and post-stroke individuals. We adapted an operant H-reflex conditioning protocol from previous work which focused on regulating soleus H-reflex, here targeting the femoral nerve innervating the quadriceps instead of the tibial nerve. We presented visual feedback of the electrically evoked RF H-reflex, which appeared as a bar graph. Participants were asked to reduce the bar height below a given threshold and were provided a running score of performance. The experiment consisted of 6 baseline sessions without feedback and 24 feedback training sessions, 2–3 sessions per week over a 3-month period. We expected down-regulation of RF H-reflex would be feasible in both healthy and post-stroke individuals. This study investigates the feasibility of a novel paradigm aimed at quadriceps spasticity management post-stroke. The potential of this intervention could lead to development of a targeted, non-pharmacological and non-invasive treatment for quadriceps hyperreflexia.

## Methods

### Subjects

A total of 7 participants [5 unimpaired (H1–H5, ages 20–26 years, 3 M/2 F), 2 post-stroke (S1–S2, 1 M/1 F see Table 1)] were recruited for this study. Each participant was able to stand for 10-min intervals unassisted, walk on a treadmill for 10-min, and provide informed consent. Exclusion criteria included: lower limb musculoskeletal injury (e.g., fractures, sprains, strains, tendonitis, or bursitis), functionally relevant weight-bearing restrictions, vision impairment and must not have taken antispasmodic medication one day prior to the session. All 7 people participated in down-conditioning. The study was approved by the University of Texas Institutional Review Board, and all subjects gave informed consent prior to participation. Both post-stroke individuals had right hemiparesis and the experiment was conducted on the right side.

**Table 1:** Clinical information of post-stroke participants.

Subject	S1	S2
Age	54	68
Gender	M	F
Years post-stroke	4	1
Type of stroke	Ischemic (embolic)	Hemorrhagic
Location of stroke	Left middle cerebral artery	Left basal ganglia
Modified Ashworth score (MAS)		
Pre	3 (knee extensor)	1 + (knee extensor)
	2 (knee flexor)	0 (knee flexor)
	3 (ankle plantarflexor)	1 + (ankle plantarflexor)
Post	2 (knee extensor)	0 (knee extensor)
	2 (knee flexor)	0 (knee flexor)
	1 + (ankle plantarflexor)	1 + (ankle plantarflexor)
Assistive device	Customized AFO	Cane, AFO, Wheelchair
Other complications	Aphasia mild cognitive impairment	

Years post-stroke is the duration from the date of stroke to the beginning of the training. Modified Ashworth Score^33^ was measured by the clinician before and after training. Both participants received therapy outside their experimental participation. MAS Key: 0 = no increase in tone, 1 = slight increase in tone (catch/release at end ROM), 1 +  = slight increase in tone, (catch/release at ½ ROM with resistance to end ROM, 2 = more marked increase in tone through ROM, passive movement easy, 3 = considerable increase in tone, passive movement difficult, 4 = affected part in rigid flexion or extension. AFO is ankle–foot orthosis. “Pre” refers to prior to training, while “Post” refers to after training.

### Acquisition and procedures

**Figure 1:**
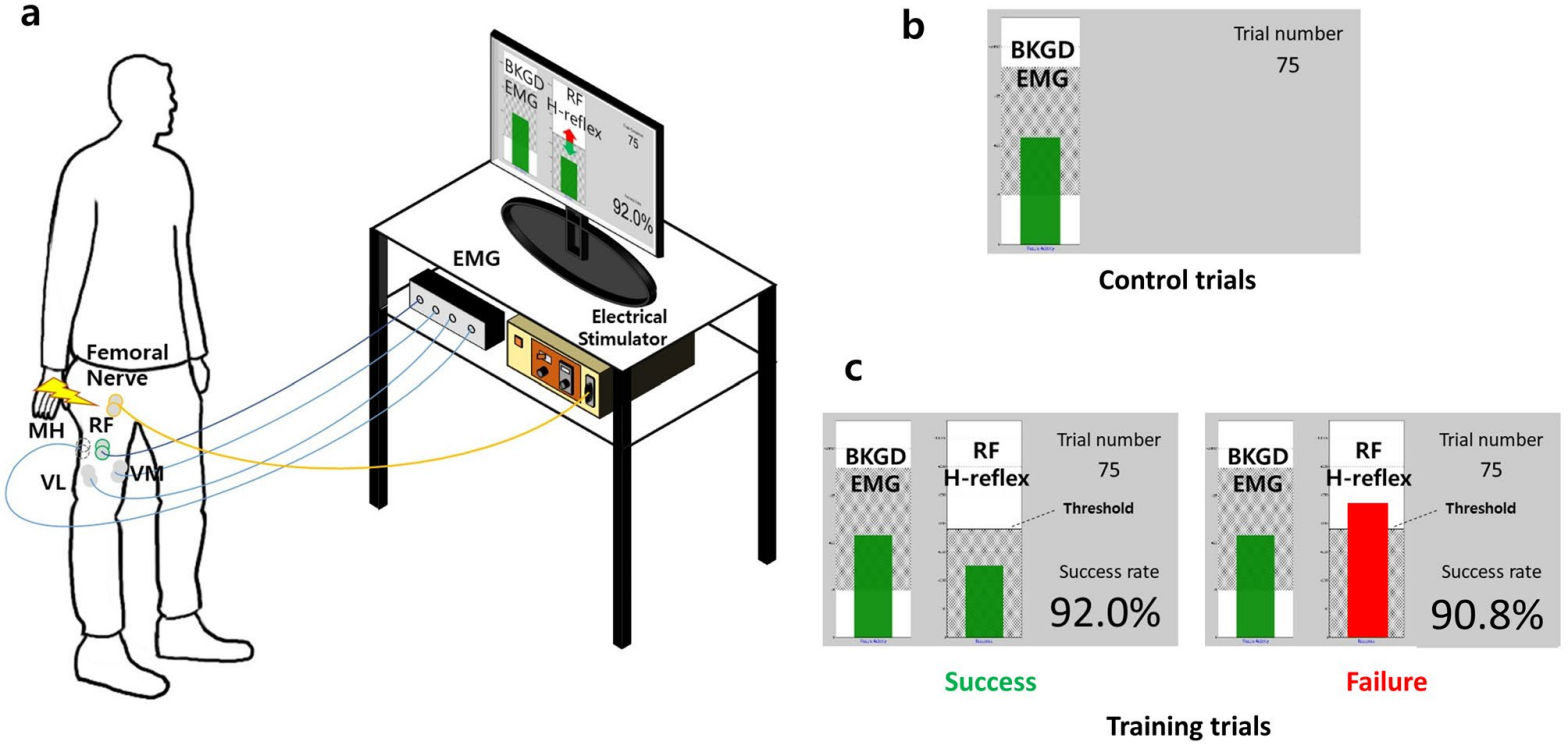
Experimental setup and visual feedback of RF (rectus femoris) operant H-reflex conditioning. (**a**) Participant with electrodes on the right leg is in the upright standing posture, facing visual feedback from a computer monitor. (**b**) During control/baseline trials, only the background EMG level of the RF was provided on the left bar graph. If the background EMG level of the RF and its antagonist pair MH (medial hamstrings) was within the shaded region, the bar remained green and the electrical stimulation was delivered. Otherwise, the bar turned red with no stimulation or trial progression. (**c**) During the training trial, RF H-reflex size and cumulative success rate were provided along with background EMG. The bar turned green if the RF H-reflex was below the threshold and turned red if larger than the threshold.

Our procedure was modeled after previous operant H-reflex conditioning experiments by Thompson, Wolpaw, and colleagues^19,25,27^. The experimental setup consisted of 8-channel surface electromyography (EMG) sensors (AMT-8, Bortec, Calgary, AL), bipolar electrodes (Ag–AgCl, Noraxon, Scottsdale, AZ) a constant current electrical stimulator (Digitimer DS8R, Hertfordshire, UK), a data acquisition board (NI-PCIe 6321, Austin, TX), a desktop computer, and EPOCS software^28^ (Fig. 1). Additionally, during assessment sessions, stroke subjects walked on an instrumented split-belt force treadmill (Bertec, Columbus, OH), during which the ground reaction forces (GRF) were recorded at 1 kHz, and lower limb kinematic data were recorded via inertial motion capture (IMU, Xsens, Enschede, Netherlands) at 60 Hz.

The experiment was conducted over 30 experimental sessions per individual, with 3 sessions per week on non-consecutive days. To prevent diurnal variation in H-reflex magnitude^29^, all training was performed at the same time of the day for each individual. Each experimental session consisted of preparation, femoral nerve navigation, recruitment and stimulation stages. The *preparation stage* started with drawing a grid on the surface of the femoral triangle on the leg. The femoral nerve was identified using an anatomical landmark-based method known as the “four-three finger technique”^30^. Surface EMG electrodes were attached over the muscle belly of the rectus femoris (RF), medial hamstring (MH), vastus medialis (VM), and vastus lateralis (VL) based on commonly used anatomical landmarks^31,32^. RF was the primary focus for H-reflex monitoring, MH was monitored for antagonist activity, and VM and VL were monitored to validate the training specificity of RF H-reflex conditioning. EMG activity was amplified, bandpass filtered (3–1000 Hz), and sampled at 1 kHz.

During the *femoral nerve navigation* stage, the optimal stimulation location of the femoral nerve was established via a monopolar probe electrode to each point on the grid drawn on the femoral triangle^34^. Electrical stimulation was delivered as a square stimulus pulse, 1 ms in duration, intensity ranging between 10 and 20 mA or above depending on the individual, to elicit a reflex response with the subject in quiet standing. We monitored the time-course of the reflex response. The H-reflex was determined as the peak response following the initial stimulus artifact (< 3 ms) and M-wave (~ 10–25 ms), typically within 30–40 ms post-stimulus. We used the following criteria to identify the optimal H-reflex response: (1) a clear biphasic shape, (2) a clear distinction from the M-wave or in case of contamination the biphasic signal lies on the downward slope of the M-wave, and (3) largest signal magnitude of all the candidate spots. After the optimal stimulation spot was located, the monopolar probe was replaced with a disposable Ag/AgCl snap electrode (circular, 1 cm diameter) and the anode electrode (square 5 × 5 cm) was placed over the surface of gluteus maximus on the ipsilateral side. To avoid session-to-session variability in electrode locations, the positions of all electrodes were marked using semi-permanent surgical skin marker. After electrodes were placed, a maximum voluntary contraction (MVC) for RF and MH were measured separately. Participants were asked to sit on a chair with their ankle fastened with a belt to the leg of the chair and knee flexed to 90°. The investigator asked the participant to extend or flex their knee with maximum effort for 5–7 s. The participant repeated this three times with 15 s intervals in between. The representative MVC was determined as the mean of three MVC trials.

During the *recruitment* stage, the RF H-reflex recruitment curve was achieved with the subject in a quiet standing posture. Electrical stimulation was delivered at 0.14 Hz only if the background EMG level of RF and MH were within a 10–20% MVC window^35^ respectively, representing a slight tonic voluntary activation to avoid altering reflex excitability. To find the maximum M-wave amplitude of the RF, we increased stimulation intensity by 2 mA increments starting at 10 mA until the RF M-wave amplitude remained constant despite increasing current in three successive stimulus intensities, defined as M_max_^36^. For each stimulus intensity, two H-reflex responses were recorded. The H-reflex size was calculated as the mean of the peak-to-peak amplitudes of the two responses. The corresponding intensity (I_max_) that elicited maximal representative H-reflex (H_max_) was used in the following trials. Post-stroke individuals did not require any additional support for standing, however for safety purposes a handrail was placed in front of the individual.

During the *stimulation stage*, also in performed in a standing posture, the femoral nerve was stimulated at I_max_ at 0.14 Hz based on the same 10–20% MVC requirement. The first 6 sessions were baseline sessions with no feedback of RF H-reflex. Each baseline session was composed of 3 runs of 75 trials, where each trial was a single stimulation pulse. The next 24 sessions were training sessions. During training, each session began with 20 control trials with no feedback, followed by 3 runs of 75 trials using the peak-to-peak magnitude of RF H-reflex as visual feedback to the participant (Fig. 1). During training sessions trials, M-wave size was monitored, and stimulus strength (I_max_) was minimally adjusted to maintain the predetermined M-wave size. Post-stroke individuals participated in an additional 2 assessment sessions, one before training and one after training sessions. In participant H4, in two of the 30 training sessions, the H-reflex showed overlap with the M-wave. In offline analysis, we used a best decaying exponential fit to obtain the H-reflex estimate as illustrated in our earlier work^3^.

### Feedback

As shown in Fig. 1c, visual feedback consisted of (1) RF and MH background EMG (left bar), (2) RF H-reflex (right bar), (3) trial number, and (4) cumulative success rate. The left bar was green if the background EMG was below activation threshold but was red otherwise. The H-reflex stimulation was delivered only when the background EMG bar was green. The participant was provided with the objective to reduce the height of the right bar, representing H-reflex amplitude, below a given threshold (shaded area in Fig. 1c). The threshold was updated for each run as the 66th percentile of the H-reflex trials from the previous run. If below the performance threshold, the right bar turned green and cumulative success rate was adjusted accordingly. If above the threshold, the bar turned red, and the success rate fell. Each trial was 7 s duration, with a single stimulation pulse stimulation of 1 ms pulse width followed by feedback for the remainder of the trial. Each session was recorded on a different non-consecutive day, up to 3 times per week. The 6 baseline sessions each consisted of 3 runs of 75 control trials (225 total), while each of 24 training sessions consisted of 20 control trials and 3 runs of 75 training trials (245 total) in standing posture. Subjects were exposed to H-reflex feedback only during the training trials, and no H-reflex feedback was given during the control trials. The participant’s score for each run was cumulative success rate ($$Scr=\frac{{N}_{success}}{{N}_{trial}}$$ %) and participants were rewarded in proportion to one’s score (i.e., $$Reward\left(\$\right)=1.0+0.05*(Scr-50)$$). If the participant was successful in achieving three consecutive success rates that were equal to or higher than 90% for all three runs, they were provided with an extra dollar as bonus, which made the maximum additional compensation amount $10.00. A rest period was provided between each run to minimize fatigue, which could affect muscular responses to stimuli^37,38^.

### Clinical assessments

The two individuals with stroke participated in pre and post training assessment sessions. Participants performed a 10 m walk test (10 MWT) with instructions to walk with maximum effort. This process was repeated 4 times and the participant’s gait kinematics were recorded using inertial motion capture. Next, participants completed the quadriceps pendulum test, which is a non-invasive biomechanical method of evaluating spasticity using gravity to provoke muscle stretch reflexes during passive swinging of the lower limb^39^. Inertial measurement units were attached on top of thigh, shank, and foot to calculate the knee joint angle during the process. This test was repeated 3 times with 30 s rest periods in between. The main outcome of this test was quadriceps reflex threshold angle (QRTA), which was the angle difference from the maximum knee extension position to the first swing excursion, the first transition period of knee flexion to knee extension. Lastly, the participant was asked to walk on an instrumented treadmill while their gait kinematics, ground reaction forces (GRF), and H-reflexes were obtained. During treadmill walking, four gait phases (heel-strike, toe-off, mid-stance, and mid-swing) were detected in real-time using online GRF data using conventional gait analysis^1^. Heel-strike was detected when GRF value exceeded a predetermined force threshold (F_th_) for the first time (0% gait cycle) and toe-off was detected with GRF dropping below the threshold (F_th_) (60% gait cycle). Mid-stance was detected as the time point when 30% of step duration, pre-calculated from one’s step length and gait speed, had elapsed from the moment of heel-strike. Mid-swing was determined in a similar manner, which was detected at the 80% gait cycle. The H-reflex was elicited and measured only when a desired gait phase was detected and occurred at least 7 s after the last H-reflex trial. Twenty H-reflexes were recorded for each gait phase. A 5 min break after each of four runs was provided to minimize the effect of fatigue on H-reflex. The main outcomes consisted of (1) knee flexion range of motion (RoM), (2) peak knee flexion velocity, and (3) H-reflex amplitude during the four different gait phases.

### Outcome measures

We normalized the H-reflex measurements. For each session, the H-reflex was normalized with the session’s maximum motor response (M-wave, M_max_), which is the most commonly used method of normalization^40^. To directly compare between session-to-session performances, each session’s H-reflex trial was averaged, normalized by M_max_, and represented as a percentage (%) of the mean H-reflex of 6 baseline sessions.

Main outcome measures of each session were the average of 225 peak-to-peak H-reflex responses. Control trials, which were conducted without feedback in the beginning of each training session, provide a window into long-term plasticity of H-reflex excitability because there is no ongoing self-regulation being attempted^19^. For each session, we compared the mean amplitude of 20 control H-reflex responses.

### Training specificity of operant H-reflex conditioning

Exploring training specificity of operant H-reflex conditioning can provide evidence that the training can be targeted for a specific muscle, which in this study was the RF. During the experiment, only RF H-reflex visual feedback was provided to the subject while two adjacent quadriceps muscles (VM and VL) were recorded without being visualized by the participant. Since VM and VL share motor innervation with RF through a branch of femoral nerve, we investigated whether the shared nerve root of the quadriceps would project a broader training effect on the quadriceps. H-reflexes of VM and VL were processed in a similar way as RF; each sessions’ normalized H-reflex trials were averaged and represented as a percentage (%) of 6 baseline sessions’ mean.

### Statistical analysis

**Figure 2:**
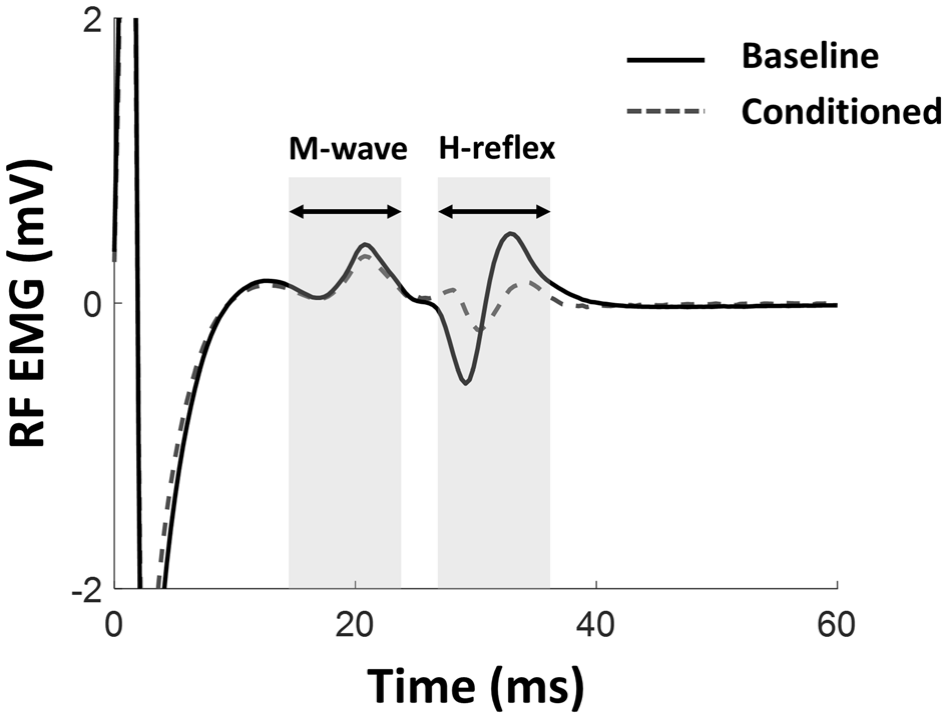
Representative RF response from participant S1 before and after training. Each shaded region represents the M-wave (15–25 ms) and H-reflex (28–38 ms). Solid black line represents averaged RF response of the baseline session and grey dashed line represents averaged RF response of the last conditioning session.

We investigated the hypothesis that RF H-reflex can be down-conditioned through operant H-reflex conditioning. We first verified normality of the data using a Shapiro–Wilk test. To validate the effect of training, a paired t-test (*a* < 0.05) was used to compare the mean of the 225 normalized H-reflex trials during 6 baseline sessions to that of the last 6 training sessions (Fig. 2). We used a linear mixed model to evaluate any spontaneous change in H-reflex during baseline, where session number was set as a fixed effect and subject number as random effect. To test for stability of M-wave size throughout training, we used a linear mixed model with session as the fixed effect and subject number as random effect. Further, we used a paired t-test to investigate corresponding changes in VM and VL H-reflex before and after training. We compared control H-reflex trials of conditioning sessions to that of baseline sessions to examine the long-term effect of training^19^ using a paired t-test. Clinical outcomes were compared before and after training using a paired t-test.

### Ethics approval

This study was conducted according to the Declaration of Helsinki and had ethical approval from the University of Texas Institutional Review Board. All subjects gave written informed consent prior to data collection.

## Results

### RF operant H-reflex conditioning

**Figure 3:**
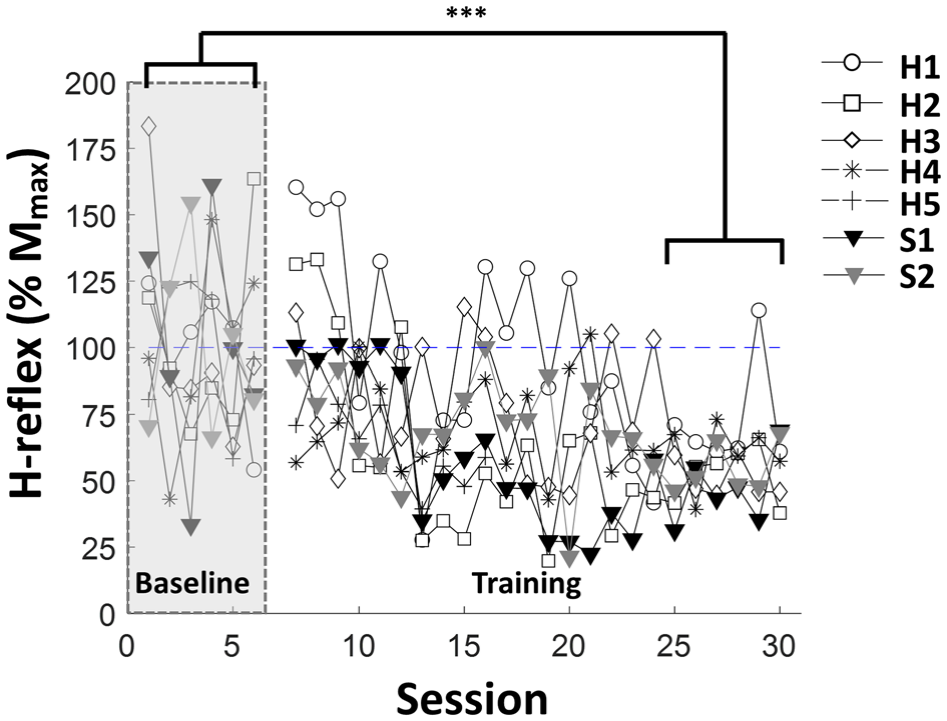
H-reflex magnitude change in RF over training. Shaded area indicates the baseline sessions and blank area indicates training sessions. Each marker represents the individuals’ (H1–H5, S1–S2) RF H-reflex means for each session. (*p < 0.05, **p < 0.01, ***p < 0.001).

All participants exhibited the ability to down-regulate RF H-reflex through training. Figure 2 illustrates a representative RF response from participant S1 before and after training. Table 2 presents the mean response of the healthy participants and individual responses of the two post-stroke participants. Because performance of post-stroke individuals was not significantly different than healthy individuals, the data was pooled for subsequent analyses. Normality was not violated in either the 6 baseline sessions (*W* = 0.9759,* p* = 0.52) or the last 6 training sessions (*W* = 0.9834,* p* = 0.85). There was no spontaneous decrease in H-reflex amplitude during baseline sessions (*F* = 0.53,* df* = 40,* p* = 0.47), compared to the training sessions (*F* = 27.18,* df* = 142,* p* < 0.0001). We observed a decrease in H-reflex amplitude when comparing the last 6 training sessions with the 6 baseline sessions (43.82 ± 4.81% (SE), *df* = 5,* p* < 0.0001). We did not find a change in M-wave amplitude across sessions (Linear mixed model, *F* = 0.003,* p* = 0.95). Figure 3 illustrates the summary statistics of H-reflex amplitude of 7 participants over each session.

**Table 2:** Summary of results based on neurological condition.

Subject	Muscle	Conditioned H-reflex (% baseline)
H1–H5	RF	58.37 ± 2.91***
	VM	52.46 ± 3.44***
	VL	71.74 ± 6.87*
S1	RF	46.87 ± 5.59*
	VM	74.62 ± 10.09ns
	VL	70.89 ± 10.55*
S2	RF	54.56 ± 3.88*
	VM	17.34 ± 9.27**
	VL	14.58 ± 0.92**

Values represent mean ± SE and are expressed as the percentage of baseline H-reflex mean. The table shows the significant differences from the six baseline sessions’ H-reflex (*p < 0.05, **p < 0.01, ***p < 0.001).

### Long-term effect of conditioning on the control H-reflex size

**Figure 4:**
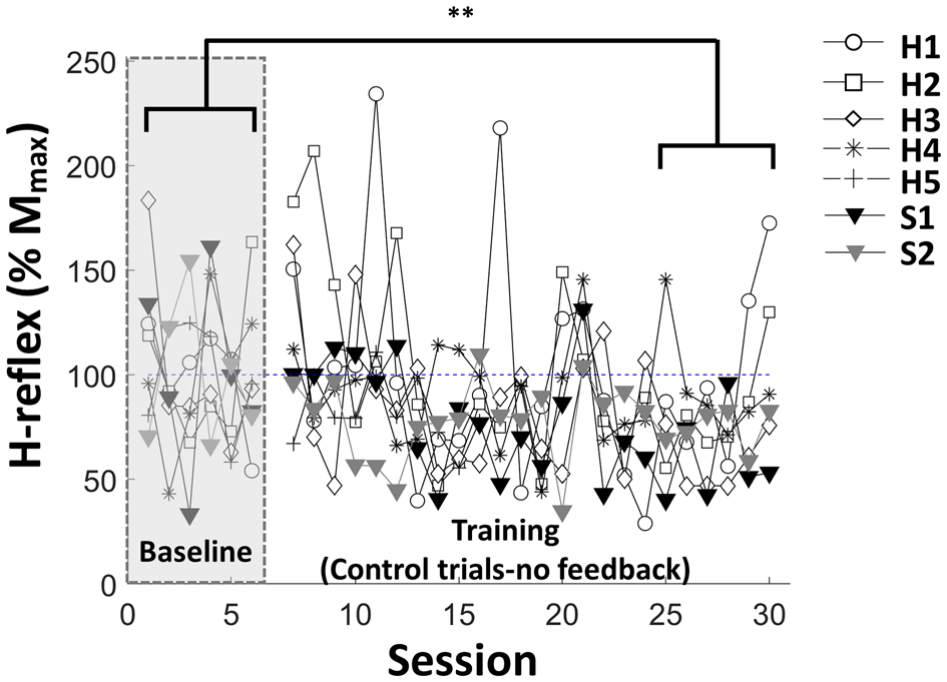
H-reflex magnitude change among control trials shows long-term effect of the training. Data during training phase extracted from 20 control trials prior to introduction of feedback for each session. Each marker represents the individuals’ (H1–H5, S1–S2) control RF H-reflex means for each session. (*p < 0.05, **p < 0.01, ***p < 0.001).

Consolidation of H-reflex down-conditioning following training can be observed in the first 20 control (no feedback) trials of each session. Figure 4 shows the time-course change of the control RF H-reflex for all participants. For the control trials (i.e., no feedback) of all 7 individuals, the change between baseline and the last 6 training sessions was (20.86 ± 5.77% (SE), *df* = 5,* p* < 0.01).

### Training specificity of RF operant H-reflex conditioning: comparison with VM and VL

**Figure 5:**
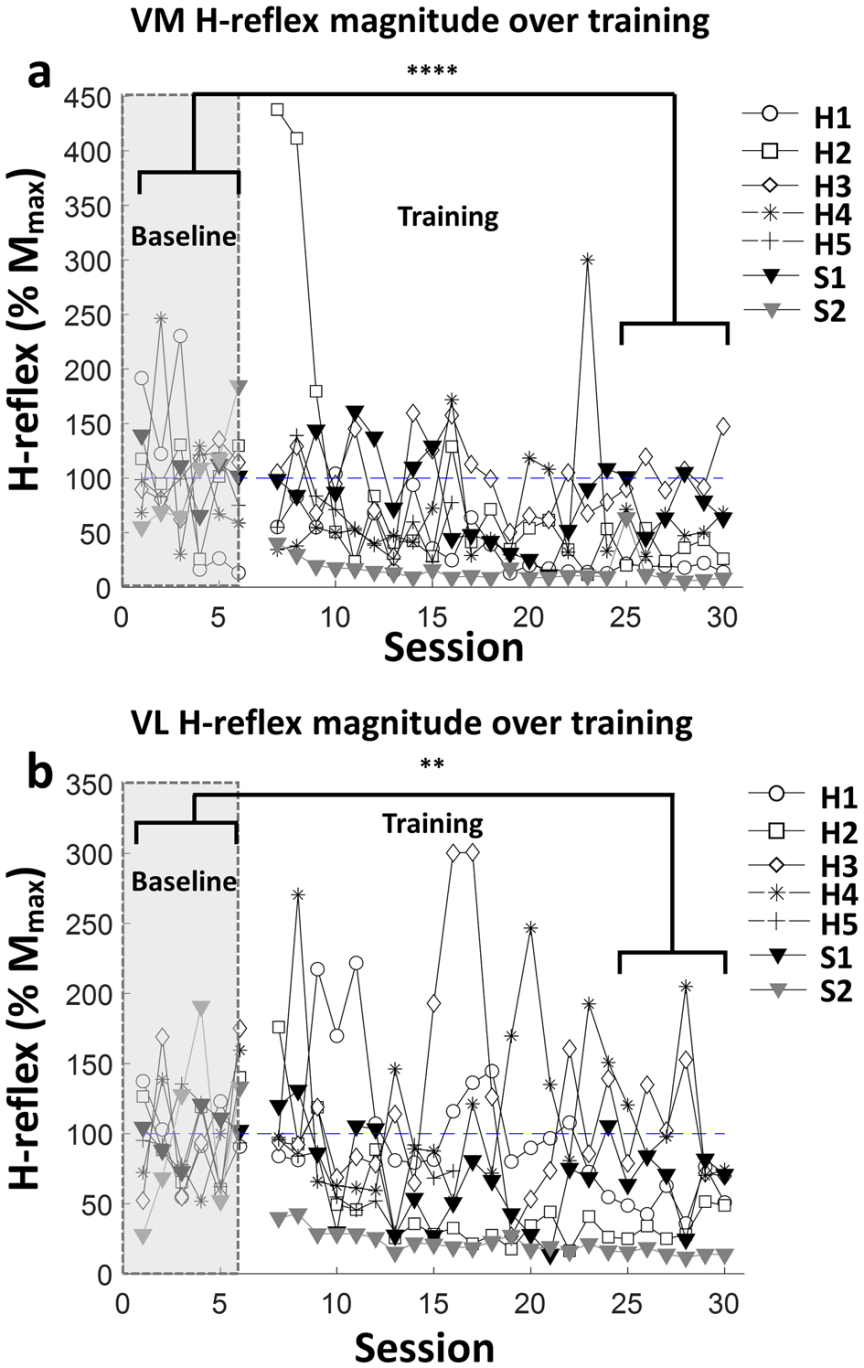
Muscle training specificity of RF Operant H-reflex Conditioning in VM (**a**) and VL (**b**) in all participants. Shaded area indicates the baseline sessions and blank area indicates training sessions. Each marker represents the individuals’ (H1–H5, S1–S2) VM and VL H-reflex means for each session. (*p < 0.05, **p < 0.01, ***p < 0.001, ****p < 0.0001).

The femoral nerve innervates all four quadriceps muscles, but feedback is only provided from one muscle, raising the question of muscle training specificity. Performance of healthy individuals separate from post-stroke individuals can be seen in Table 2. For healthy individuals, H-reflex decreased significantly for both VM and VL, whereas mixed result was monitored among post-stroke individuals. We pooled these results in Fig. 5 for VM (49.16 ± 5.42% (SE) drop, *df* = 5,* p* < 0.0001) and VL (37.24 ± 7.37%(SE) drop, *df* = 5,* p* < 0.001), respectively.

### Behavioral effect of the RF operant H-reflex conditioning in post-stroke individuals

We conducted pre/post training assessments with the two post-stroke individuals. Knee flexion RoM and peak knee flexion velocity significantly improved both during overground and treadmill walking for S1. However, for S2, the improvement in knee flexion kinematics was limited to RoM during overground walking. Table 3 illustrates the results. Quadriceps spasticity, quantified as QRTA, improved in S1 by 10.55° (*df* = 3,* p* = 0.016), while S2 had a statistically insignificant change of 3.50° (*df* = 3,* p* = 0.536). Reflex modulation (Table 3) for S1 improved significantly for all four gait phases, where the mean reduction was 19.21%. In S2, H-reflex excitability decreased significantly in three gait phases (heel-strike, toe-off, and mid-stance), with a mean decrease of 23.4%.

**Table 3:** Intervention effect on gait kinematics.

S1			S2		
Measurement	Pre-assess	Post-assess	Measurement	Pre-assess	Post-assess
QRTA (deg)	32.47 ± 3.47	42.92 ± 2.96*	QRTA (deg)	41.81 ± 0.7	45.31 ± 5.18^ns^
Knee flexion RoM (°)			Knee flexion RoM (°)		
(Overground)	12.27 ± 0.36	14.40 ± 0.26****	(Overground)	44.59 ± 0.77	51.05 ± 0.54****
(Treadmill)	6.57 ± 0.40	10.10 ± 0.39****	(Treadmill)	46.15 ± 1.13	46.53 ± 1.20^ns^
Pk vel. (°/s)			Pk vel. (°/s)		
(Overground)	226.83 ± 4.54	264.64 ± 5.81****	(Overground)	154.46 ± 6.69	179.38 ± 18.78^ns^
(Treadmill)	138.01 ± 6.73	190.59 ± 5.95****	(Treadmill)	146.93 ± 9.36	140.22 ± 6.28^ns^
Gait phase H-reflex			Gait phase H-reflex		
Heel-strike	33.63 ± 0.92	19.68 ± 0.52****	Heel-strike	96.27 ± 6.56	60.28 ± 4.42****
Toe-off	47.44 ± 2.94	17.41 ± 0.76****	Toe-off	102.65 ± 5.58	68.63 ± 1.11****
Mid-stance	31.69 ± 2.38	18.97 ± 0.42****	Mid-stance	80.43 ± 2.45	54.71 ± 2.76****
Mid-swing	37.05 ± 2.87	16.89 ± 0.60****	Mid-swing	78.95 ± 11.09	81.66 ± 3.95^ns^

Values represent mean ± SE of Quadriceps Reflex Threshold Angle (deg), knee flexion RoM (deg), and peak-velocity (deg/s) during overground (10 MWT) walking and treadmill walking. The table shows the significant differences between the pre-assessment and post-assessment session (*p < 0.05, **p < 0.01, ***p < 0.001, ****p < 0.0001).

## Discussion

The goal of this study was to investigate the feasibility of self-modulation of RF H-reflex excitability in humans. We trained 7 individuals (5 healthy and 2 post-stroke), over 24 sessions. Our results provided the first evidence that RF H-reflex can be down-regulated, and we did not observe any difference in ability to down-regulate in 2 post-stroke individuals compared to healthy participants. The down-regulation of RF generalized to other quadriceps muscles (VM and VL). The 2 post-stroke individuals exhibited improvements in reflex modulation generalized over the gait cycle and gait performance. We conclude that RF H-reflex down-conditioning is feasible in humans and its effects should be evaluated in a controlled clinical trial on post-stroke individuals.

Our findings suggest that individuals were successful in down-regulating their RF H-reflex via operant H-reflex conditioning. Comparing the H-reflex amplitude in the six baseline sessions to the last six training sessions resulted in a 42% drop in healthy individuals, 49% drop for the two post-stroke individuals, and 44% drop across all 7 individuals. Previous animal model research indicated that cerebrospinal tract (CST) transmission was critical for operant H-reflex learning^26^ suggesting that those with reduced CST integrity resulting from stroke may not be able to learn. Our findings indicate that damaged corticospinal pathways due to stroke do not prevent successful learning. Our results may also imply improved inhibition of reflex activity via brainstem pathways such as the cortico-reticulospinal tract^6,7^. It is also notable that H-reflex response decreases with age^41^, and therefore one might expect reduced ability to further down-regulate in the older post-stroke participants. However, other pioneering work recently showed the ability of post-stroke individuals to self-modulate H-reflex of the soleus, albeit with a 50% non-responder rate^27^. A controlled trial remains to be conducted that examines the effect of damaged pathways on operant learning and performance.

We expected to observe successful down-conditioning of RF since previous work has shown successful soleus H-reflex down-conditioning^19,24^. However, in neural operant conditioning, even across modalities, approximately 30% of individuals are non-learners^19,24,42^. Although only 7 individuals were included in our cohort, there were no non-responders, suggesting the potential for a robust operant conditioning paradigm. Our analysis of 6 baseline sessions showed no evidence of spontaneous decrease in H-reflex amplitude that could be mistaken as down-conditioning. Further, there is no evidence of spontaneous decrease in H-reflex amplitude over sessions in previous work^19^. Thus, it is highly unlikely that RF H-reflex spontaneously decreased over time. Additionally, the 44% drop in RF H-reflex performance during training compares favorably to previous research of operant conditioning of soleus H-reflex in healthy and spinal cord injured individuals, averaging a 31% drop^25^. This suggests that not only is operant RF H-reflex training feasible, but it is also at least as feasible as the more established operant soleus H-reflex conditioning.

Trials conducted without feedback, control trials, provide a window into long-term plasticity. Overall, we observed a decrease in all individuals (21 ± 5.77(SE) %), where the drop in post-stroke individuals (S1 = 25%, S2 = 40%) was more evident than that of the healthy group (16%). These results align with earlier findings, where the drop of control H-reflex of the soleus was larger in participants with spinal cord injury (24%) than healthy individuals (16%)^25^. This difference is attributed to the hypothesis of negotiated equilibrium^43^, the concept that neural circuitry already behaving well will not be affected by neuromodulation, but neurologically affected circuitry will.

We found that operant conditioning based on femoral nerve stimulation generalized to other innervated quadriceps muscles. Additional monitoring of adjacent quadriceps muscles (VM and VL) was performed to validate the specificity of RF operant H-reflex conditioning. A significant drop in VM and VL H-reflexes in healthy individuals between the first and last 6 sessions was demonstrated. Although not conclusive, these results suggest that operant conditioning based on stimulation of a peripheral nerve that innervates multiple muscles may affect the entire muscle group. It should also be noted that cross-talk between muscles may also have affected these results. Animal studies by Chen et al.^44^ found that operant conditioning of the soleus affected quadriceps H-reflex excitability, and as such the generalization of operant conditioning to multiple muscles, especially innervated by the same nerve, was expected. The degree of generalization will indicate the specificity of the effect of training and needs to be characterized further.

We observed functional changes in gait in the post-stroke individuals following training. These results align with earlier studies that have shown functional improvement following the operant H-reflex conditioning. Chen et al.^29^ have shown rats with incomplete spinal cord injury (SCI) improved gait asymmetry following the training. The studies by Thompson et al. and Manella et al.^24,25^ have shown gait improvements (i.e., speed, endurance, less clonus, easier stepping etc.) in people with SCI. Tahayori and Koceja^45^ and Thompson et al.^27^ have separately shown post-stroke individuals who were able to down-regulate their soleus H-reflex correlated with movement improvements. In our study, S1 exhibited improvements in knee ROM and knee flexion velocity in both overground and treadmill walking conditions, but S2 only showed improvements in knee kinematics only in overground walking. Both participants exhibited better improvements during overground walking than treadmill walking. Overground walking was measured during 10 m walk test, where the participants were asked to set their speed with the maximum effort. No wiring from EMG sensors or the stimulator was attached, and a belt type harness was worn. In contrast, during treadmill walking, participants had to walk in a fixed and self-selected speed that was predetermined, the wiring from EMG sensors and stimulator was present, a full-body harness had to be worn, and no gait support devices (i.e., cane, rolling walker) were allowed. Thus, it is possible that the differences between overground and treadmill walking could be due to the experimental setup. Clinical and laboratory measurements of hyperreflexia showed improvements in both post-stroke individuals. For QRTA, only S1 exhibited an improvement. In both S1 and S2, H-reflex amplitude during different gait cycle phases decreased, suggesting generalization of training during standing to walking^20^. However, we should note that we did not measure M_max_ for every gait phase, so it is possible that some of the decrease in H-reflex could be attributed to changes in stimulus efficacy. While no conclusions can be drawn based on the changes in 2 post-stroke individuals, these results validate the feasibility of RF H-reflex down-conditioning for post-stroke individuals with SKG and quadriceps hyperreflexia. A clinical trial to further investigate the effectiveness of this intervention in this population is warranted.

This study investigates the feasibility of RF H-reflex down-conditioning based on 7 individuals, 2 of them post-stroke. One of the main limitations of this study is the small sample size (7). Despite the small sample, the effect size (3.85) and statistical power (98.7%) were substantial and suggest that a larger sample size would not likely alter our conclusions on the feasibility of the procedure in humans. Due to training only 2 post-stroke individuals, we cannot generalize the feasibility in operant conditioning of RF H-reflex conditioning to a larger post-stroke population. However, it is noteworthy that these two post-stroke individuals with different levels of impairment were able to down-regulate their RF H-reflex at least as well as their unimpaired counterparts (Table 2). It was previously questioned whether post-stroke individuals were physiologically capable of such self-modulation^26^. While S2 demonstrated improvement in H-reflex during most gait phases except mid-swing, her functional improvement was not as pronounced as S1. For S2, the pathway lesion (cortico-basal ganglia-thalamo-cortical loop) was different than S1 (corticospinal) resulting in a different underlying mechanism of change. Future investigations that expand understanding the relationship between H-reflex excitability and clinical outcomes would be of value. Future research may also investigate how age and amount of time since stroke, including the development of spasticity and weakness, may also influence the efficacy of the procedure. In this experiment we extended existing protocols and offered monetary incentives for performance^19^. Such incentivization is common in operant conditioning^46^, but would not be likely in a clinical context.

## Conclusions

Our observations suggest that RF H-reflex down-conditioning is feasible in humans and generalizes to other quadriceps muscles. The successful training of 2 post-stroke individuals provides promise that the ability to regulate H-reflex may extend to this neurologically impaired population, however a larger sample size is needed to confirm the intervention’s efficacy, long-term plasticity, and validate clinical outcomes. These initial results provide insight for alternative treatments of spasticity that may lead to a non-pharmacological intervention in post-stroke Stiff-Knee gait and other spasticity-related disorders.

## Data Availability

The datasets generated during and/or analyzed during the current study are available from the corresponding author on reasonable request.
